# Sleep improvement strategies for people with vision impairment: a scoping review

**DOI:** 10.1136/bmjopen-2025-113100

**Published:** 2025-12-24

**Authors:** Benedict Leonard-Hawkhead, Mapa Prabhath Piyasena, Tunde Peto, Gianni Virgili, R M A van Nispen, Katie Curran

**Affiliations:** 1 Centre for Public Health, Queen’s University Belfast, Belfast, UK; 2 School of Medicine, Faculty of Health, Medicine and Social Care, Anglia Ruskin University Vision and Eye Research Institute, Cambridge, UK; 3 University of Florence, Florence, Italy; 4 Eye clinic, AOU Careggi Teaching Hospital, Florence, Italy; 5 Ophthalmology, Amsterdam UMC Locatie VUmc, Amsterdam, The Netherlands

**Keywords:** OPHTHALMOLOGY, SLEEP MEDICINE, EPIDEMIOLOGY, PUBLIC HEALTH

## Abstract

**Abstract:**

**Objectives:**

To explore existing strategies for managing sleep disorders in individuals with vision impairment (VI), identifying interventions, geographical trends and research gaps.

**Design:**

Scoping review.

**Data sources:**

Medline ALL (Ovid), Embase and Web of Science Core Collection, with supplementary searches in Google Scholar. The final search was completed on 28 November 2025.

**Eligibility criteria for selecting studies:**

Original research studies examining strategies to manage sleep disorders in adults (≥18 years) with VI, published in English. Studies focusing on animal models or unrelated to sleep management were excluded.

**Data extraction and synthesis:**

Two reviewers independently screened titles, abstracts and full texts using Covidence, extracted data using a predefined form and resolved discrepancies by consensus. A narrative synthesis approach was used to summarise findings by intervention type, study design and outcomes.

**Results:**

Of 4368 records screened, 16 studies met inclusion criteria. Participants ranged from 18 years to 85 years (median 40.5). Most studies included individuals with no light perception, though VI definitions were often inconsistent. Pharmacological interventions dominated (13/16, 81.3%), mainly melatonin or melatonin receptor agonists, with some use of zopiclone, low-dose benzodiazepines and tricyclic antidepressants. Non-pharmacological approaches were under-represented, including bright light exposure (n=1), virtual Hatha yoga (n=1) and caffeine modulation (n=1). Substantial variation existed in sleep assessment methods.

**Conclusions:**

This scoping review highlights the predominant focus on pharmacological treatments, especially melatonin, while non-pharmacological strategies remain underexplored. Future research should explore accessible, non-pharmacological interventions and address sleep health inequities faced by individuals with VI.

**Registration:**

10.17605/OSF.IO/7E83R.

**STRENGTHS AND LIMITATIONS OF THIS STUDY**

- Comprehensive scoping review conducted using a transparent, protocol-driven approach (registered with Open Science Framework).
- Systematic searches across multiple databases and grey literature sources ensured broad coverage.
- Variation in definitions and exclusion of non-English studies may limit findings.
- As a scoping review, formal assessment of study quality and intervention effectiveness was not undertaken.

## Introduction

 Sleep constitutes a crucial aspect of human health, essential for physical, mental and emotional well-being.^1^ However, the impact of sleep disorders poses a significant challenge to individuals’ overall quality of life, particularly when compounded by pre-existing health conditions. Vision impairment (VI) introduces an additional layer of complexity, as it can disrupt the body’s natural circadian rhythms, which are biological processes that follow an approximately 24-hour cycle and regulate sleep-wake patterns, hormone release and other physiological functions.^2 3^ These rhythms are primarily synchronised to the external environment by light exposure, which is detected by intrinsically photosensitive retinal ganglion cells (ipRGCs) in the eye and transmitted to the suprachiasmatic nucleus (SCN) of the hypothalamus, the body’s central circadian pacemaker.^4^ The SCN regulates the secretion of melatonin from the pineal gland, with levels typically rising in the evening to promote sleep and decreasing in response to morning light exposure.^5^

In individuals with total blindness, the absence of light perception prevents the circadian system from aligning with the 24-hour day, leading to free-running circadian rhythms.^3^ This disruption may result in Non-24-Hour Sleep-Wake Disorder (N24SWD), where the internal sleep-wake cycle progressively shifts out of sync with the societal schedule.^6^ Such misalignment often causes irregular sleep patterns, excessive daytime sleepiness and impaired functioning, which significantly affect quality of life.^7^ N24SWD is particularly prevalent among those with no light perception (NLP).^3 8 9^ Additionally, those with partial vision loss might experience varying degrees of sleep disturbances, influenced by the extent of their remaining light perception and the functionality of their ipRGCs.^10^ The severity and type of VI therefore directly impact the extent of circadian disruption, potentially complicating the maintenance of a regular sleep schedule.^11^ Beyond circadian rhythm disturbances, individuals with VI and eye disease are also at higher risk of experiencing other sleep disorders. Conditions such as insomnia, hypersomnia (excessive daytime sleepiness) and obstructive sleep apnoea have been reported at elevated rates in this population, each arising through distinct mechanisms.^12 13^

Sleep disorders are closely associated with negative outcomes for mental health and well-being.^1^ Individuals with VI are particularly vulnerable, with studies showing elevated levels of anxiety and depression compared with the general population.^2^ This increased anxiety may further disrupt sleep through heightened arousal and worry, while poor sleep can, in turn, exacerbate anxiety symptoms, creating a bidirectional cycle that negatively impacts mental health. Among individuals with VI, these challenges may also intensify feelings of social isolation or dependency.^14^ Poor sleep can worsen cognitive functions such as memory, attention and decision-making, thereby affecting work performance and daily activities.^15^ Moreover, chronic sleep deprivation may contribute to long-term health issues such as cardiovascular disease and diabetes, further diminishing overall quality of life.^16^

Recognising the negative impact of sleep disorders on individuals with VI, this scoping review aims to map the existing body of evidence, identify research gaps and explore strategies to improve sleep quality.

## Methods and materials

A protocol for this scoping review was registered to Open Science Framework in July 2024. The scoping review adhered to the Preferred Reporting Items for Systematic reviews and Meta-Analyses (PRISMA) extension for Scoping Reviews guidelines.

### Search strategy

Our search strategy includes systematic exploration across multiple electronic databases including Medline ALL (Ovid), Embase and Web of Science Core Collection to gather literature on managing sleep disorders in people with VI. Additionally, Google Scholar was used to review the initial 200 results, aligning with Haddaway *et al*’s recommendations for comprehensive coverage.^17^ We expanded our search to inspect the references of included studies to ensure a comprehensive identification of applicable literature.

### Key search terms

The search strategy for this review was carefully developed with guidance from an information specialist (RF) at Queen’s University Belfast (QUB) to optimise the search process. Search terms were iteratively refined and tailored for each database to enhance the retrieval of relevant literature on managing sleep disorders in individuals with VI. The number of records identified per database is detailed in online supplemental table S1.

### Inclusion criteria

- All studies focusing on adults (>18 years old) with VI, including NLP.
- Research on strategies for addressing sleep disorders (all types), including interventions and management techniques in humans.
- Publications in English with no restrictions on publication date.
- Original research only

### Exclusion criteria

- Studies not relevant to sleep disorders or strategies for people with VI.
- Non-English publications.
- Animal studies.

While our review does not restrict inclusion to studies that use formal classifications of VI, we recognise and emphasise the value of standardised definitions (ie, WHO definition for VI) in enhancing study design and comparability. Our decision to adopt a broader definition was driven by the practical need to include a wider range of research. Limiting our scope to formally classified cases would have significantly reduced the available literature.

### Process of study selection

A research fellow (KC) from QUB conducted the database searches, exporting titles and abstracts as RIS files, which were then imported into Covidence for screening and duplicate removal. A second reviewer (BLH), a PhD researcher from QUB, also performed independent screening. Full texts were reviewed for eligibility by both reviewers, with disagreements resolved through discussion or adjudication by a third reviewer (MPP) if needed.

**Figure 1:**
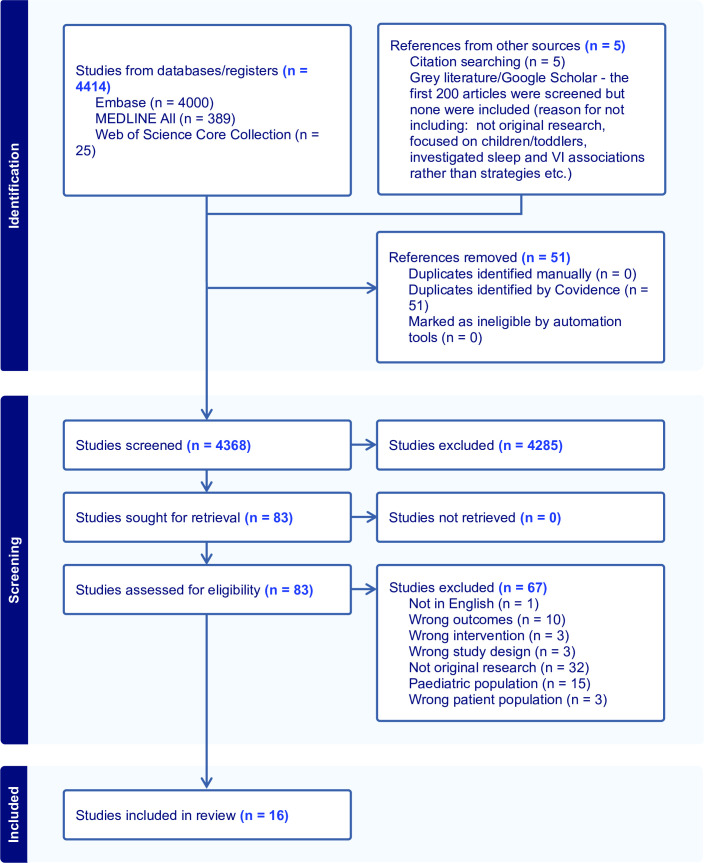
Preferred Reporting Items for Systematic reviews and Meta-Analyses flow diagram of sleep interventions in people with vision impairment.

The initial search was conducted on 18 March 2024 and updated on 28 November 2025 (online supplemental table S1). The updated search retrieved 564 records from Embase, 25 from MEDLINE ALL and three from the Web of Science Core Collection for the period 2024–2025. All Embase records were excluded at title and abstract screening. Of the 25 records identified in MEDLINE ALL, three were assessed at full-text review but subsequently excluded. Of the three records identified in the Web of Science Core Collection, one was considered potentially relevant at title and abstract screening but was subsequently excluded. No additional studies met the inclusion criteria. The study selection process for the initial search is detailed in the PRISMA flow diagram (figure 1).

### Data collection and charting

Relevant data on study design, sample characteristics, interventions and outcomes were extracted using a predefined Excel-based form (online supplemental table S2). Two reviewers (KC, BLH) extracted and organised the data according to key themes and issues, resolving discrepancies through discussion until consensus was reached.

### Summarising the evidence

A narrative synthesis approach, as recommended by Popay *et al*, was used to systematically analyse and interpret findings across studies. This involved identifying key themes related to the management of sleep disorders in individuals with VI. The frequency and types of interventions were examined to provide a comprehensive overview of current management strategies. This approach was well-suited to capturing the breadth of the topic and identifying gaps in the literature. To ensure the reliability and validity of the synthesis, an evaluation was conducted using the Joanna Briggs Institute critical appraisal tool. This included an assessment of population characteristics such as age, gender and severity of VI.

## Results

A total of 4368 studies were screened, 83 underwent full-text review and 16 met inclusion criteria. Reasons for excluding studies are outlined in figure 1.

All studies were from high-income countries, mainly the USA (n=7) and UK (n=3), with single studies from Germany, Israel, Sweden, Finland and New Zealand. A large multicentre study included 27 US and 6 German sites. This highlights a research focus in North America and Europe, with limited global representation (table 1).

**Table 1:** Summary table of included studies

Variables	Study details (n=16)
Study year (range)	1988–2024. Only one study from 2015 to 2024 has been published
Study types (n, %)	
Case studies	3 (18.8)
Observational studies	3 (18.8)
Longitudinal observational study	2 (12.5)
Single blind, placebo-controlled crossover trial	1 (6.3)
Randomised controlled trial	1 (6.3)
Quasiexperimental studies	1 (6.3)
Double-blind, placebo-controlled crossover trials	3 (18.8)
Experimental single-blind design	1 (6.3)
Observational prevalence study	1 (6.3)
Sample size (n*,*range)	1–469
Age range (min–max age, years)	18–85 years, median 40.5 years
Gender distribution (M, range %)	7.69–100%
Main interventions studied:	
1. Melatonin or melatonin receptor agonist	
0.5 mg, melatonin	3 (18.8)
1 mg, 2 mg, 4 mg, melatonin	1 (6.3)
5 mg, melatonin	5 (31.3)
10 mg, melatonin	2 (12.5)
20 mg, tasimelteon	1 (6.3)
2. Psychoactive substances including zopiclone, low dose benzodiazepines and tricyclic antidepressants	1 (6.3)
3. Lighting intervention (3300 lux)	1 (6.3)
4. Caffeine intervention (150 mg)	1 (6.3)
5. Virtual Hatha yoga	1 (6.3)
Mode of delivery:	
Oral administration (melatonin, caffeine)	13 (81.3)
Telephone survey (medication adherence)	1 (6.3)
Online yoga (for relaxation)	1 (6.3)
Light therapy	1 (6.3)
Countries represented:	
USA	7 (43.6)
UK	3 (18.8)
Germany	1 (6.3)
Finland	1 (6.3)
Sweden	1 (6.3)
Multicentre study (27 sites in the USA and six sites in Germany)	1 (6.3)
Israel	1 (6.3)
New Zealand	1 (6.3)

Most studies (n=11, 68.8%) included participants who were totally blind, while others had varying levels of VI, from reduced light perception to moderate and severe impairment. One case study described a participant simply as having ‘loss of sight’, highlighting inconsistencies in how VI is defined. Terms like ‘blindness’ and ‘light perception’ were often used without reference to standardised criteria such as those from the WHO, limiting comparability across studies. Adopting WHO definitions and clearly reporting levels and types of VI would enhance reliability and support analysis. Eye conditions varied widely, including retinitis pigmentosa, age-related macular degeneration (AMD) and rarer disorders like Leber congenital amaurosis, reflecting the diversity of visual challenges studied.

Of the 16 included studies, nine different study designs were used. Crossover trials (n=4)^18^,^21^ were most common, followed by observational (n=3),^22^,^24^ longitudinal (n=2)^24 25^ and case studies (n=3).^26^,^28^ Others included a single-blind crossover trial,^21^ one randomised controlled trial (RCT),^29^ one quasiexperimental study^30^ and a prevalence study^31^ (table 1).

Gender representation was inconsistently reported across studies. Of the 16 studies, seven (43.8%) reported outcomes only for male participants, while nine (56.3%) included both genders. Among the mixed-gender studies, male representation ranged from 33.3% to 90%. One study did not specify gender. This variation in reporting and the exclusion of females in several studies highlights the need for more balanced and transparent recruitment and reporting strategies in future research (table 1).

A variety of methods were used across the 16 studies to assess sleep quality and circadian rhythms. Melatonin levels, measured through plasma, saliva or urine were commonly assessed, often alongside cortisol levels. Body temperature was occasionally used as an additional physiological marker. In contrast, objective sleep assessments such as polysomnography (a comprehensive sleep assessment) and actigraphy (wrist-worn devices tracking sleep-wake cycles) were less frequently used. Subjective measures were also reported, with four studies using validated questionnaires such as the Pittsburgh Sleep Quality Index (PSQI), and four studies incorporating sleep logs or diaries to capture self-reported sleep patterns (table 2).

**Table 2:** Objective and subjective sleep measures

Measures	Frequency	Details of assessment
Objective measures:		
Melatonin levels (plasma, saliva, urine)	9	Includes plasma melatonin, saliva melatonin, 6-sulfatoxymelatonin (aMT6s) and urinary melatonin
Body temperature	3	Includes oral measurements for circadian rhythm analysis
Cortisol levels (plasma, urine)	4	Measures stress-related hormones via plasma and urinary samples
Polysomnography	2	Used for sleep-wake patterns and melatonin level assessment
Actigraphy	1	Objective measurement of sleep-wake cycles
Adrenocorticotropic hormone	1	Measurement of adrenocorticotropic hormone related to cortisol production
Subjective measures:		
Sleep logs	4	Used for monitoring sleep patterns and duration over time
Validated questionnaires	4	Includes Pittsburgh Sleep Quality Index and Stanford Sleepiness Scale (one time) for sleep quality, and Beck Anxiety Inventory (BAI) for anxiety assessment
Qualitative data	2	Case histories and mood ratings to assess behavioural and emotional states

Across the 16 studies, primary outcomes varied, with circadian rhythm entrainment assessed in 8/16 (50%) studies. Sleep disturbance reduction was evaluated in three studies (18.8%), while 13 studies (81.3%) reported additional outcomes such as circadian phase shifts, hormonal alignment, sleep quality and daytime functioning (online supplemental table S3).

### Pharmacological interventions

Of the 16 studies, 13 (81.3%) focused on pharmacological interventions, with melatonin being the most commonly investigated treatment for stabilising circadian rhythms and improving sleep quality in individuals with VI. Melatonin, a hormone produced by the pineal gland, regulates the body’s sleep-wake cycle. Dosages varied across studies, with 5 mg daily being most common (n=5), typically administered 1 hour before bedtime or at a fixed time (eg, 22:00). Lower doses such as 0.5 mg (n=3) and variable doses ranging from 1 mg to 4 mg (n=1) were also evaluated.

Several studies reported that melatonin effectively entrained circadian rhythms and improved sleep quality, particularly among individuals with NLP. Correct timing of administration was emphasised, with doses taken 1–2 hours before the desired bedtime (typically 0.5–10 mg) consistently supporting circadian rhythm alignment (online supplemental table S3).

Tasimelteon (20 mg, n=1), a melatonin receptor agonist, was evaluated as an alternative treatment for N24SWD in totally blind individuals (table 1). The SET (Safety and Efficacy of Tasimelteon) and RESET (Randomized-withdrawal study of the Efficacy and Safety of Tasimelteon to treat Non-24-Hour Sleep-Wake Disorder (Non-24)) trials demonstrated its sustained effectiveness, with up to 90% of participants remaining entrained even after treatment was withdrawn. This was the only study to assess tasimelteon’s impact on sleep outcomes in this population.^29^

A prevalence study highlighted that melatonin was infrequently prescribed, with other medications such as zopiclone, low-dose benzodiazepines and tricyclic antidepressants more commonly used.^31^

### Non-pharmacological interventions

Non-pharmacological interventions included bright light exposure (15 min–1 hour daily, n=1),^32^ virtual Hatha yoga sessions (n=1)^30^ and caffeine intake assessments (150 mg at 10:00 am, n=1).^27^ Home-based virtual Hatha yoga reduced sleep disturbances and anxiety (significant improvements in sleep quality and anxiety).^30^ Bright light therapy (3300 lux) improved subjective sleepiness and mood, but did not achieve entrainment in blind individuals, while caffeine did not entrain circadian rhythms, though it improved alertness and mood (online supplemental table S3).

## Discussion

This scoping review highlights the complex relationship between VI and sleep disorders, emphasising the diversity of study designs, interventions and measures used to investigate this topic. The study underscores the limited geographic scope of research on sleep interventions for VI, which has so far been confined to high-income countries. Despite the high and growing prevalence of VI in low- and middle-income countries (LMICs), these regions remain underrepresented in the existing literature. Given that individuals in LMICs are disproportionately affected by VI, there is a need to expand research efforts to these areas.^33 34^ Addressing this gap could help reduce health inequalities and provide relevant interventions that may improve sleep quality and overall well-being for visually impaired individuals in underserved regions.

A recent scoping review by Choi *et al* found that several eye conditions, including blindness, glaucoma, diabetic retinopathy, AMD, retinitis pigmentosa and more, are associated with sleep problems and circadian rhythm disruptions.^12^ While their review highlighted the need for further work on non-pharmacological interventions, it did not explore existing strategies in detail.^12^ In contrast, our review examined both pharmacological and non-pharmacological approaches to managing sleep problems in individuals with VI.

Pharmacological treatments dominated the evidence base, with melatonin being the most frequently studied. Although melatonin is effective in stabilising circadian rhythms, outcomes depend heavily on timing and dosage. Despite evidence, surveys show low prescription rates of melatonin in blind individuals, with only 4% of those with reduced light perception being prescribed it.^31^ More qualitative studies to assess real-world use and barriers to accessing melatonin are needed. The inclusion of newer agents such as tasimelteon reveals ongoing exploration for optimal treatments.^29^ Tasimelteon, approved by the Food and Drug Administration in 2014 and European Medicines Agency in 2015 for treating N24SWD in adults, offers promise for individuals with total blindness. However, its high cost and limited availability pose significant accessibility challenges, with access and coverage varying across countries.^29^

Non-pharmacological interventions, including bright light therapy and Hatha yoga, were under-represented in the literature, highlighting an opportunity for further research into sustainable, accessible approaches for individuals with VI. Cognitive Behavioural Therapy for Insomnia (CBT-I) is an evidence-based treatment recommended by NICE for insomnia.^35^ Although face-to-face delivery remains the gold standard, limited availability has prompted growing interest in digital CBT-I programmes such as Sleepio, which offer scalable alternatives and have shown benefits in the general population. However, these tools have not been specifically designed or evaluated for people with VI, and accessibility barriers may limit their use.^35^ Further research is warranted to determine how digital CBT-I platforms could be adapted to meet the needs of people with VI.

Vision-specific eHealth interventions are emerging. For example, E-nergEYEze, a CBT-based and self-management-based programme developed for adults with VI, has shown good usability and acceptability in early studies.^36 37^ Although primarily designed to reduce fatigue, it includes a dedicated sleep module targeting behavioural and circadian factors, suggesting potential for adaptation to support sleep health in this population.

The studies included in this review employed a diverse range of objective and subjective measures to assess sleep and circadian rhythms, reflecting the multifaceted nature of sleep disturbance in VI. Objective tools such as polysomnography, actigraphy and hormonal assays provide valuable physiological insights, whereas subjective tools such as the PSQI and sleep diaries capture patient-reported outcomes. Combining these approaches offers a holistic understanding of sleep health but requires careful consideration of participant burden and accessibility, particularly for those with VI. This methodological diversity illustrates the complexity of the field, yet the limited number of RCTs highlights a continuing gap, especially for non-pharmacological interventions.

### Strengths and limitations

This review has several limitations. As a scoping review, the objective was to map and synthesise existing evidence rather than to evaluate the quality or effectiveness of interventions; therefore, no formal risk-of-bias assessment or meta-analysis was conducted. The included studies were highly heterogeneous in design, population characteristics and outcome measures, which restricted direct comparison across findings. Differences in the definitions and classifications of VI were particularly notable, with few studies using standardised criteria such as those recommended by the WHO. This inconsistency makes it difficult to interpret outcomes by severity or type of impairment.

Detailed demographic reporting was also limited in many studies, particularly for gender, socioeconomic status and ethnicity, constraining understanding of subgroup differences and potential health disparities. The underrepresentation of women in some studies further reduces the generalisability of findings. Finally, publication and language bias may have influenced the evidence base, as only English-language studies were included.

Despite these limitations, this review highlights several important knowledge gaps. First, there is a clear need for well-designed studies evaluating both pharmacological and non-pharmacological interventions specifically in people with VI. Second, mechanisms linking VI, circadian rhythm disruption and sleep quality remain poorly understood and warrant further physiological and longitudinal investigation. Finally, the accessibility and effectiveness of digital or behavioural sleep interventions for this population should be explored through co-designed and inclusive research approaches. Addressing these gaps will be essential to inform evidence-based strategies that improve sleep and overall well-being in individuals with VI.

## Conclusions

While pharmacological treatments such as melatonin and tasimelteon can entrain circadian rhythms, their success is limited by individual responsiveness, timing and accessibility. Non-pharmacological approaches, including light therapy and CBT-I, offer promising, person-centred alternatives that align with patient preferences. Larger, well-designed RCTs are needed to evaluate the effectiveness and feasibility of tailored, inclusive, non-pharmacological interventions. Standardisation of VI classification, ideally using WHO criteria, will enhance comparability across studies. Future research should also prioritise diverse populations across geographic and economic contexts, including LMICs, and ensure inclusive recruitment strategies, particularly regarding gender balance, to improve the generalisability and real-world relevance of findings.

## Supplementary material

10.1136/bmjopen-2025-113100 online supplemental file 1

## Data Availability

All data relevant to the study are included in the article or uploaded as supplementary information.
