# Supplementary material for: Sleep improvement strategies for people with vision impairment: a scoping review
Source: BMJ Open. 2025 Dec 24;15(12):e113100. doi: 10.1136/bmjopen-2025-113100 (PMC12742078; doi:10.1136/bmjopen-2025-113100)
Supplement: online supplemental file 1 [file bmjopen-15-12-s001.docx]

Table S1: Search terms and hits from three main databases

| **SEARCHES** | | |
| --- | --- | --- |
| **Embase** (<1974 to 2024 March 18>) | **Medline All** (1946 to March 18, 2024) | **Web of Science Core Collection**  (All fields, no date restriction) |
| 1. exp visual disorder/  2. visual impairment/ or visually impaired person/  3. exp sleep disorder/  4. insomnia/  5. wakefulness/  6. circadian rhythm disorder/  7. circadian rhythm sleep disorder/  8. non 24 sleep wake cycle.mp.  9. exp sleep apnea syndromes/  10. sleep apnoea.mp.  11. intervention*.mp.  12. treatment*.mp.  13. exp phototherapy/  14. support group/  15. device approval/  16. medical device/  17. strateg*.mp.  18. 1 or 2  19. 3 or 4 or 5 or 6 or 7 or 8 or 9 or 10  20. 11 or 12 or 13 or 14 or 15 or 16  21. 18 and 19 and 20  22. conference abstract/  23. 21 not 22  24. article/  25. 23 and 24  26. limit 25 to (human and English language)  **Total =** **4000** | 1. exp Vision Disorders/ 2. Visually Impaired Persons/ or vis* impair*.mp. 3. Visual Acuity/ or light perception.mp. 4. Rod Opsins/ or Retinal Ganglion Cells/ or Retina/ or Melanopsin.mp. 5. Melanins/ 6. exp Sleep Wake Disorders/ 7. insomnia.mp. 8. Wakefulness/ or Sleep/ or Circadian Rhythm/ or non 24 sleep wake cycle.mp. 9. exp Sleep Apnea Syndromes/ 10. sleep apnoea.mp. 11. 1 or 2 or 3 or 4 or 5 12. 6 or 7 or 8 or 9 or 10 13. 11 and 12 14. intervention*.mp. 15. treatment*.mp. 16. exp Phototherapy/ 17. support group*.mp. 18. Device Approval/ or medical device*.mp. 19. strateg*.mp. 20. 14 or 15 or 16 or 17 or 18 or 19 21. 13 and 20   **Total =** **389** | 1. "vis* impair*" or blindness or "low vision" or "light perception" or "registered blind" 2. "sleep disorder*" or insomnia or "sleep quality 3. intervention* or treatment* or "light therap*" or cbt 4. human* not (animal* or mouse or mice or rats)   **Total = 27** |
| * An updated database search was conducted on 28 November 2025. This identified 564 records in Embase, 25 in MEDLINE ALL, and 3 in the Web of Science Core Collection for the period 2024-2025. All Embase records were excluded at title and abstract screening. Of the 25 records identified in MEDLINE ALL, three were assessed at full-text review but subsequently excluded. Of the three records identified in the Web of Science Core Collection, one was considered potentially relevant at title and abstract screening but was subsequently excluded. No additional studies met the inclusion criteria. | | |

*Table S2: Predefined Data Extraction Form*

| **Study Identification** | | **Study Design** | **Population Characteristics** | | **Sample Characteristics** | | **Interventions*** | | | **Outcomes** | | | | **Additional Information** | | |
| --- | --- | --- | --- | --- | --- | --- | --- | --- | --- | --- | --- | --- | --- | --- | --- | --- |
| Authors | Publication Year | Type of Study (e.g. RCT, observational study, qualitative study) | Age range of participants | Severity of VI | Sample size | Recruitment methods | Description of Sleep Interventions | Duration and frequency of interventions | Mode of delivery (e.g., in person or online) | Primary and secondary outcomes related to sleep disorders | Measurement tools used for outcomes | Follow-up duration | Study location | | Setting (clinical, community, home-based) | Other potential factors to influence sleep (societal, other co-morbidities) |
| Data on interventions were extracted using the Population–Concept–Context (PCC) framework, as shown in Table S3 | | | | | | | | | | | | | | | | |

Table S3: Population, Concept, Context (PCC) Framework: Interventions for Sleep Disturbances in People with VI

| **Study** | **Population (P)** | **Concept (C) (Type of Sleep Intervention)** | **Context (C)** | **Outcome Measures** | **Effectiveness** |
| --- | --- | --- | --- | --- | --- |
| Adrent 1998 [5] | Not specified but states 'lost his sight' (n=1) | 5mg melatonin at 2300 for 2 weeks and placebo for the last 2 weeks | Clinical | Reduced sleep disturbances and entrainment of circadian rhythms | Melatonin helped regulate circadian cycle and enhanced well-being. |
| Dowling 2023 [30] | Vision impairment (varied severity) (n=13) | Virtual Hatha yoga sessions | Home-based | Reduction in sleep disturbances (Sleep quality, PSQI) and anxiety (BAI) | Lower PSQI scores post-intervention indicates improved sleep quality (P = 0.002) Reduced anxiety (P = 0.002) |
| Fischer 2003 [18] | Totally blind (n=12) | Single 5 mg dose of melatonin administered 1 hour before bedtime (pharmacological level) | Clinical (sleep lab) | Total sleep time/efficiency and ACTH and cortisol rhythms during sleep | Participants reported feeling more well-rested, balanced, and relaxed after melatonin versus placebo (*p < 0.05* for all). |
| Hack 2003 [19] | Totally blind (n=10) | In a placebo-controlled, single-blind design, subjects received 0.5 mg melatonin or placebo (identical lactose-filled capsule) daily at 2100 for at least one full circadian beat cycle according to their baseline aMT6s  period (range of treatment: 26-81 days) (i.e., the number of days to complete one full cycle, 360 degrees). All  subjects received both treatments. | Clinical | Entrainment of circadian rhythms (assessed via urinary cortisol and aMT6s rhythms) Subjective sleep quality and nap frequency/duration (assessed via daily sleep and nap diaries) | - 6 of 10 (60%) blind participants entrained to a 24-hour circadian rhythm with 0.5mg melatonin. - 1 (10%) participant showed a shortened period but did not fully entrain. - 3 (30%) participants did not entrain, possibly due to melatonin administration occurring in the phase delay portion of the circadian timing. |
| Lewy 2001 [22] | Totally blind (N=3) | A de nova dose of 0.5mg melatonin could entrain free-running circadian rhythms. 1-2 hours before preferred bedtime | Clinical | Entrainment of circadian rhythms | All three (100%) participants successfully entrained to the 24-hour cycle with 0.5 mg melatonin alone (1-2 hours before bedtime). Longer duration than 3 weeks may be required for full entrainment. |
| Lewy 2004 [23] | Totally blind (n=7) | Daily low-dose melatonin administration (0.5 or 0.05mg) | Clinical | Entrainment of circadian rhythms | All 7 (100%) participants successfully entrained to a 24-hour cycle with low-dose melatonin (0.5 mg or 0.05 mg), even though treatment was initiated on the delay zone of the circadian cycle. |
| Lewy 2001 [25] | Totally blind (n=8) | 10mg melatonin at fixed bedtime | Laboratory with controlled administration | Circadian Entrainment (to a 24-hour cycle), based on melatonin onset (MO) in plasma. Phase Angle of Entrainment (PAE): the interval between the time of melatonin intake and melatonin onset. Correlation between pretreatment circadian period (tau) and PAE. | - 8 out of 9 (88.9%) totally blind individuals were successfully entrained to a 24-hour rhythm with 10 mg melatonin. - The only person who did not entrain had the longest pretreatment tau (24.9 h). - Strong correlation found between pretreatment tau and PAE (r = 0.76, P < 0.05). - Individuals with longer taus required smaller PAE (took melatonin closer to melatonin onset) to entrain. - After entrainment, MOs consistently occurred at predictable intervals relative to dosing time, confirming stability. |
| Lockley 2000 [21] | Totally blind (n=7) | Five of the seven subjects were treated daily for one full circadian cycle with 5 mg melatonin or placebo (identical lactose-filled gelatine capsule) at 2100 h. The remaining two subjects also received melatonin for a full circadian cycle, but these data were compared with a no-treatment baseline | Laboratory with controlled timing | Entrainment of circadian rhythms assessed via phase shifts in aMTGs and cortisol’s | - 3 of 7 (42.9%) participants entrained to a 24-hour rhythm during 5 mg melatonin daily at 21:00. - 4 of 7 (57.1%) participants did not entrain, maintaining a free-running rhythm.   Successful entrainment was associated with timing of melatonin administration closer to the advance phase. Melatonin can entrain some blind individuals, but not all, and timing matters. |
| Lockley 2015 [29] | Totally blind (n=104) | Tasimelteon 20mg versus placebo | Clinical (sleep lab) | Entrainment of circadian rhythms | **SET Trial:**   - 20% entrained in the tasimelteon group vs 3% in placebo (P = 0.0171) - 24% showed clinical response vs 0% in placebo (P = 0.0028). **RESET Trial:** - 90% remained entrained on tasimelteon vs 20% on placebo after withdrawal (p = 0.0026).   Tasimelteon significantly improves and maintains entrainment in totally blind individuals with non-24 disorder. |
