# Supplementary material for: Sleep improvement strategies for people with vision impairment: a scoping review
Source: BMJ Open. 2025 Dec 24;15(12):e113100. doi: 10.1136/bmjopen-2025-113100 (PMC12742078; doi:10.1136/bmjopen-2025-113100)
Supplement: Supplementary file 2 [file bmjopen-2025-113100.reviewer_comments.pdf]

PEER REVIEW HISTORY

BMJ Open publishes all reviews undertaken for accepted manuscripts. Reviewers are asked to complete a checklist review form and are provided with free text boxes to elaborate on their assessment. These free text comments are reproduced below.

ARTICLE DETAILS

Title (Provisional)

Sleep Improvement Strategies for People with Vision Impairment: A Scoping Review

Authors

Leonard-Hawkhead, Benedict; Piyasena, Mapa Prabhath; Peto, Tunde; Virgili, Gianni; van Nispen, R. M.A.; Curran, Katie

---

VERSION 1 - REVIEW

**Reviewer**	**1**
**Name**	**Crossland, Michael**
**Affiliation**	**UCL Institute of Ophthalmology**
**Date**	**25-Nov-2025**
**COI**	**None**

I am content with the changes made and congratulate the authors on this important work.

**Reviewer**	**2**
**Name**	**Vijayakumar, Venugopal**
**Affiliation**	**National Institute of Naturopathy**
**Date**	**26-Nov-2025**
**COI**	**None**

All the previous concerns raised has been appropriately addressed.

Thank you
